# “Why not ask the doctor?” Barriers in help-seeking for sexual problems among older adults in Poland

**DOI:** 10.1007/s00038-020-01472-6

**Published:** 2020-09-04

**Authors:** Gabriela Gore-Gorszewska

**Affiliations:** Jagiellonian University, Cracow, Poland

**Keywords:** Ageing, Sexual health, Help-seeking behaviour, Physician–patient relations, Qualitative research

## Abstract

**Objectives:**

Existing data show that older adults rarely seek medical or psychological help for their sexual problems. The current study explores the barriers in help-seeking faced by older adults from a conservative Central European country.

**Methods:**

Thirty semi-structured interviews were conducted among Polish residents (16 women, 14 men) aged 65–82. The data were analysed thematically, with coding validity and analytical rigour ensured throughout the process.

**Results:**

Three main barriers in seeking help were identified: not recognising sexual problems; fear for the doctors’ disapproval; lack of knowledge how to access appropriate services. The data reveal that the participants’ main concern is that health providers would dismiss their problems as trivial. Older adults from Poland suffer from the lack of fundamental knowledge about their sexual functioning.

**Conclusions:**

Employing qualitative methodology to understand why older adults from conservative cultures do not seek help for their sexual problems might contribute to existing literature by providing evidence from different cultural settings, and help to develop and implement appropriate interventions. Implications related to health providers’ attitude towards older patients’ concerns are further discussed.

**Electronic supplementary material:**

The online version of this article (10.1007/s00038-020-01472-6) contains supplementary material, which is available to authorised users.

## Introduction

Many older adults consider sexuality an important part of their life and continue sexual activity well into late adulthood (Laumann et al. 2006; Hinchliff et al. 2018). Despite sexual expression and sexual health now being recognised as an unarguable aspect of quality of life and well-being at all life stages, the literature indicates that sexual health in later life is not integrated into standard healthcare (Gott et al. 2004; World Health Organization 2006; Lindau and Gavrilova 2010). Older adults report numerous barriers to discussing sex-related issues in a healthcare context, while physicians tend to consider older patients’ sexuality as outside their domain (Malta et al. 2018; Gewirtz-Meydan et al. 2019). It leads to a relatively small number of older adults receiving medical or psychological treatment for their sexual problems (Lindau et al. 2007; Moreira et al. 2008; Bauer et al. 2016). This is considered a growing problem, as unaddressed sexual health issues may negatively affect the sex-lives of ageing generations (Malta et al. 2018).

The concept of sexual health includes—although is not limited to—the absence of disease or dysfunction that reduces the possibility of having pleasurable sex (WHO 2006). However, how sexual problems are defined varies from the strict criteria of the biomedical perspective to various subjective meanings related to difficulties experienced during sexual encounters (Mitchell et al. 2011; American Psychiatric Association 2013). This can be associated with how an individual understands sex, its purpose, and expression; the literature suggests this is complex and nuanced in older adults (Loe 2004; Potts et al. 2006; Marshall 2012; Gewirtz-Meydan et al. 2019; Gore-Gorszewska 2020). Hence, whether a symptom is considered a sexual problem and how it impacts the quality of sex life is personal and context dependent. Therefore, the definition for sex-related issues used in this study was purposively broad and equated them with any physiological or psychological difficulties experienced by an individual in relation to sexual activity, regardless of meeting diagnostic criteria for sexual dysfunction (Mitchell et al. 2011).

The barriers that older patients experience in discussing sexual health in medical contexts are of growing scientific interest. Older adults frequently report that being embarrassed or ashamed when initiating a conversation with a doctor about a sexual topic is a barrier to seeking help (Corona et al. 2013; Fileborn et al. 2017). Experiencing these feelings in this context is commonly associated with older individuals conforming to pervasive stereotypes about “asexual old age”. Also, the tendency of older adults to minimise the seriousness of sexual problems often leads them to feel anxious about their doctor’s reaction if a sexual issue is raised during a consultation (Gott and Hinchliff 2003; Moreira et al. 2008; Bauer et al. 2016; Fileborn et al. 2017). The anticipated attitude of the health practitioner towards sex-related topics, together with their demographic characteristics (doctor’s gender, age, etc.), has proven to be of vital importance in older patients’ decisions to initiate a discussion or not (Sarkadi and Rosenqvist 2001; Gott and Hinchliff 2003; Rutte et al. 2016; Fileborn et al. 2017). The literature points to a vicious circle of abrogating responsibility: both doctors and older adults expect the other to take the initiative and open up a discussion about sex-related issues, invoking numerous arguments to support their claims (Hinchliff and Gott 2011; Dyer and das Nair 2013; Bauer et al. 2016; Rutte et al. 2016; Malta et al. 2018; Sinković and Towler 2019).

While quantitative studies explore the general patterns as to why older adults do not seek medical help for sexual problems, their ability to provide detailed insight into the underlying reasons is limited. Unfortunately, the qualitative studies published on this topic to date focus either on clinical groups of patients without regard to their age (e.g. cancer or cardiological patients), or have been conducted among older adults in only a limited number of countries (mostly Australia, the USA, and the UK) (Dyer and das Nair 2013; Bauer et al. 2016; Sinković and Towler 2019). This could limit how transferable the findings are, due to the specificity of the participants’ social and cultural contexts (Hinchliff 2016).

One of the factors contributing to the specific sociocultural context of Polish older adults’ sexuality is the post-World War II period, during which the current older generation was growing up. Catholic Church cemented its influential position as the adversary of the Communist regime (Ediger 2005). The collective influence of the regime (e.g. suppressing the effects of the 1960s sexual revolution, considered a demoralizing capitalist influence) and traditional catholic teaching (imposing heteronormativity, double standards, conservative family values, etc.) provided little room for sex-positive attitudes (Ingbrant 2020). Sexual education for adolescents was rudimentary, oriented around parenthood, family life and biological aspects of human reproduction (Woźniak 2015), while for the adults was focused on procreation and motherhood, with a few not easily accessible exceptions of handbooks on sex and eroticism (Ingbrant 2020). Such a sociocultural context left current older generation with very limited opportunities for understanding own sexual functioning, enjoying their sex life or achieving sexual freedom (Stankowska 2008).

Sexual problems are often interconnected with other health issues in older age; they can also negatively impact older individuals’ physical, emotional, and relational well-being (e.g. self-esteem, sexual satisfaction, successful ageing) (Loe 2004; Corona et al. 2010; Træen et al. 2017; Hinchliff et al. 2018). Experiencing sex-related problems may affect the subjective sexual quality of life, may also translate to lower overall life satisfaction (Woloski-Wruble et al. 2010; Forbes et al. 2016). Considering that, it is important to recognise and properly address sexual problems holistically within the public health arena. This study aimed to qualitatively investigate barriers to discussing sex-related issues during medical consultations among older adults in the specific cultural setting of Central Europe (Poland). This knowledge could be valuable to healthcare providers and policy makers when planning/implementing effective preventive or educational interventions designed to enhance the quality of older generations’ sexual life and well-being.

## Methods

This paper is based on the findings from a qualitative, interview-based study conducted among 30 Polish residents (16 women, 14 men), of various educational backgrounds and socio-economic statuses (Table 1), aged 65–82 (*M *= 71.4, SD = 5.24). Participants were recruited through posters (with an invitation to contact the interviewer) distributed to health centres, pharmacies, and retirement communities in two locations in southern Poland. Purposive sampling was used to increase the sample’s diversity regarding age, area of residence, and relationship status. Candidates were provided with details about the study’s scope and its procedures, given assurances about anonymity and confidentiality measures, as well as informed of their right to withdraw at any time.

**Table 1:** Sample characteristics (*N *= 30) (Poland 2019)

Characteristics	Total *N* = 30		Women *n* = 16		Men *n* = 14	
	*n*	%	*n*	%	*N*	%
*Age categories*						
65–69	12	40.0	7	43.75	5	35.7
70–74	9	30.0	5	31.25	4	28.6
75–82	9	30.0	4	25.0	5	35.7
*Marital status*						
Single	3	10.0	2	12.5	1	7.15
Divorced	11	36.7	2	12.5	9	64.3
Widowed	11	36.7	10	62.5	1	7.15
Married	5	16.6	2	12.5	3	21.4
*Relationship status*						
No partner	15	50.0	8	50.0	7	50.0
New relationship	11	36.7	6	37.5	5	35.7
Long-term relationship	4	13.3	2	12.5	2	14.3
*Sexual health (self*-*assessed)*						
Very poor/poor	6	20.0	4	25.0	2	14.3
Moderate	19	63.3	10	62.5	9	64.3
Good/excellent	5	16.7	2	12.5	3	21.4
*Education*						
Primary	1	3.3	0	0	1	7.15
Secondary/vocational	18	60.0	11	68.8	7	50.0
Tertiary/higher	11	36.7	5	31.2	6	42.9
*Employment*						
Retired	20	66.7	13	81.25	7	50.0
Semiretired	6	20.0	2	12.5	4	28.6
Employed	4	13.3	1	6.25	3	21.4
*Place of residence*						
Rural	3	10.0	1	6.25	2	14.3
Small/medium town	8	26.7	6	37.5	2	14.3
City	19	63.3	9	56.25	10	71.4
*Age*, *M* (SD)	71.4 (5.24)		70.5 (4.89)		72.5 (5.59)	

Semi-structured, face-to-face interviews were conducted by the author (psychologist, sexologist) between January and May 2019 after obtaining the participants’ informed consent and took place in their place of choosing (their house or the author’s office). Each interview lasted two to three hours and was audio recorded. A friendly, conversational atmosphere was maintained to ensure a feeling of security, and to establish the trust essential to discussing sensitive topics. Being part of a larger study exploring the sexuality of older Polish individuals, the interview guide was broad, and addressed a number of topics related to the participants’ sexual life (Table 2). After the interview, participants completed a brief demographic form.

**Table 2:** Interview topics guide (Poland 2019)

	Interview topics^a^
1.	*Opening questions* Social background and situation
2.	*Childhood and adolescence* Closeness (emotional, physical) in the family of origin Emotional climate concerning body, nakedness, and display of affection in the family of origin Sexual education in the family of origin, at school
3.	*Sexual experiences throughout life* Participants’ sexual history and current sexual life Sexuality and own body today
4.	*Beliefs and attitudes towards sexuality* The meaning of sex now and in the past The importance of sex and its role in life now and in the past Participants’ attitudes and beliefs regarding sexuality in later life
5.	*Health and illness* Participants sexual health/sexual problems (currently and in the past) Sexual health in later life in relation to public health services
6.	*Closing questions*

^a^The interview guide was adapted from the Healthy Sexual Aging Study, courtesy of prof. Bente Træen

During the debriefing, none of the interviewees expressed discomfort or distress; on the contrary, many found it gratifying, comforting or meaningful. According to the participants, the interviewer’s comparatively young age (30 +) did not inhibit their disclosure. Female participants admitted that the researcher’s gender (female) facilitated open discussion, while male participants explained their honesty and openness by stressing out that they considered the interview a discussion with a professional researcher, not a woman. Three of the interviews conducted were not included in the final analysis as answers to the research questions were not obtained. In order to ensure confidentiality during the data collection and analysis, no third party was present during the interviews, no personal details were kept after the interviews, the author anonymised the recordings before transcription, and only the author had access to all the transcribed interviews (kept on a password-protected computer). Ethics approval for the study was granted by The Philosophy Department’s Research Ethics Committee of the Jagiellonian University.

**Fig. 1:**
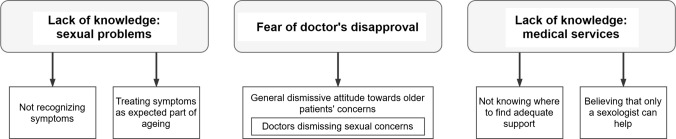
Barriers to consulting sexual problems with healthcare providers, identified in the participants’ narratives (Poland 2019)

The qualitative data analysis adhered to the principles of thematic analysis (Braun and Clarke 2006) and followed the steps required to ensure the quality of the analysis and the trustworthiness of its findings. The author familiarised herself with the data through multiple readings of a number of transcripts (*n *= 10), the coding process then followed. The initial coding categories encompassed the *barriers to seeking help* previously identified in the literature, and the notions that emerged from the interviews (deductive and inductive approach). A subsample of the transcripts (*n *= 5) was open-coded by an independent researcher (a psychologist, experienced in qualitative methodology) to ensure the validity of the coding. Differences between the two coding outcomes were resolved through discussion. The codes were collated and combined into a number of themes, then reviewed for internal homogeneity and external heterogeneity (Braun and Clarke 2006). The reviewed themes were organised into a thematic map, which was then applied to the remaining transcripts. Additional in vivo codes identified in the process were then reviewed and, if applicable, used to modify the already defined themes, forming the final thematic map (Fig. 1). MAXQDA software was used for all data analysis. Analytical rigour was maintained throughout the recurrent discussions between the author and independent researcher in order to meet the methodological standards for qualitative research (National Institute of Health and Care Excellence 2012), and a clear audit trail was kept in step-by-step analysis notes. To ensure the richness of the data and to allow the reader to evaluate the elements of the analysis and its interpretative conclusions, the participants’ direct quotes have been provided (Sinković and Towler 2019). The quotations were translated by the author and verified by professional translating services. A table with the main coding categories and illustrative quotations is provided in Supplementary Table 1.

## Results

Three main themes were identified in the participants’ narratives as being barriers to seeking help: (1) lack of knowledge or understanding of sexual problems; (2) fear for doctors’ disapproval; and (3) lack of knowledge about appropriate medical services. No gender differences were observed in relation to the themes presented below.

### Lack of knowledge: sexual problems

A number of participants equated sexual problems with erectile dysfunction (ED), answering akin to Wendy, sexually inactive widow: “*In my marriage, we had none of the problems you ask about. His [penis] was always erect and ready”* (Wendy, 73, married for 42 years). Several female and male participants admitted that they were unaware of the existence of any sexual problems other than ED, and needed examples to engage in this part of the interview. If, however, sexual problems were recognised, they were often seen as normal in later life and rarely considered as requiring treatment: *“I’m old, what should I expect*—*to be fully functional? Of course, a lot doesn’t work. It is normal at my age”* (Maria, 69, long-term marriage). Comparable comments were expressed by men regarding ED, and by women regarding vaginal dryness, pain during penetration, or a general lack of sexual desire. It is noteworthy that many older women in this study admitted experiencing these sexual problems from a young age, and always considered them a normal part of sexual functioning. The perception that sexual problems were inherent in later life led many participants to passively accept the status quo and the certainty that nothing could be done to change the situation, even if notable discomfort present. This often translated into claims that consulting a doctor would be futile and pointless, such as in case of Adam, who experiences pain during occasional intercourse: *“And what’s he going to do? This is how it’s always been and how it must be”* (Adam, 72, divorced, single).

### Fear of doctor’s disapproval

Excluding past contexts of pregnancy or procreation, all the participants reported never being asked by a doctor—general physician (GP) or specialist—about their sexual health or sexual problems. When prompted further, the participants explained that in their younger days it was obvious and understandable for them, as sex was a taboo subject within their society. Anna, who remains intimate with her husband but ceased to engage in sexual intercourse after menopause, explained: *“No way! I couldn’t make myself talk to a friend, not to mention a doctor. Anyway, in those times you wouldn’t want to bother a doctor with such a silly issue while he had other seriously ill patients to take care of”* (Anna, 75, long-term marriage). This and similar statements suggest that when the participants were younger, in addition to sexuality being taboo, sexual problems were typically considered less important than other medical conditions, therefore, not worth mentioning during consultations.

Following from this, both female and male participants believed that currently, health providers would still be uninterested in their sexual problems, although for different reasons: *“I think that the sex*-*related problems of an older person are not real problems for doctors. They tell us we are lucky to walk independently, or not require surgery like so many of our peers. This is as high as we can aim apparently”* (Barbara, 68, divorced, new relationship). Barbara successfully resumed sexual activity in her new relationship at the age of 62 but, at the time, did not feel treated with respect when searching for medical support. Similarly, many participants shared the strong conviction that health providers would dismiss their sex-related problems as being trivial (“the least of your concerns”). In some cases this claim was based on assumptions made about doctors’ attitudes; however, several participants other than Barbara drew on discouraging past encounters: *“When I asked him [a cardiologist] if I should be worried about my high blood pressure in a sexual context, he replied, ‘Mr X, you should be happy that you can still climb the stairs to your apartment on the 2nd floor. Let’s focus on that, shall we?’ It was so humiliating! I’ll never ask again”* (Joseph, 75, divorced, new relationship). For Joseph, it was the first and, as he claims, the last time he brought up a sex-related concern with a doctor, which puts him at risk due to his cardiological condition.

Other participants—both women and men—elaborated on how they had felt rebuked by their doctors for raising non-essential issues during consultations: *“I asked my GP about something that was bothering me. He smiled politely and said I shouldn’t worry about it right now […] And it wasn’t even a sex*-*related question I asked him. It seems that we should be glad we’re still alive and never ask for a better quality of life, not to mention sex life”* (Dana, 66, divorced, single). According to the participants, doctors—directly or otherwise—indicate which health problems should be prioritised and disregard the rest, even if the patient considers it important and wishes to discuss it. Such experiences of feeling dismissed by a doctor, and the anticipation of such behaviour, were highlighted by the participants as particularly discouraging from raising sex-related issues during consultations.

### Lack of knowledge: medical services

The third main reason for not seeking help for sexual problems, according to this study, is twofold: a lack of knowledge about where to find relevant support, and the certainty that only a sexologist could address sexual concerns. When asked: “Where would you seek help, who would you prefer to talk to about a sexual problem?”, both female and male participants hesitated and expressed strong uncertainty: “*I have no idea. A doctor, who specialises in these illnesses maybe? Or someone like you? I honestly do not know”* (Wendy, 73, widow, single). The total lack of awareness about where to seek help that emerged from the participants’ narratives was frequently accompanied by feelings of helplessness and defeatism: *“I tried to ask a specialist, I went through all the health centres where my other doctors are located, but I just couldn’t find anyone. I didn’t know what else to do”* (John, 69, widower, new relationship). In John’s case, his ED became a problem for him after meeting a new romantic partner with whom he wanted to have sexual intercourse. Despite his efforts, John was unable to find a doctor who, in his understanding, would address his condition, and eventually gave up. Even urologists or gynaecologists were rarely associated with sexual problems, but rather with the treatment of common medical conditions (e.g. screening for prostate or cervical cancer).

Participants frequently commented explicitly that sexual problems can only be addressed by sexologists, who are hardly available: *“I often go through the lists of doctors in health centres […] I don’t think I’ve ever seen a label ‘sexologist* –*ever. And it’s obvious that such a specialist should deal with sex*-*related problems, not a GP or a gynaecologist. All doctors have their speciality, right?”* (Anna, 75, long-term marriage). Such presumptions have emerged from female and male participants’ narratives as a factor that effectively prevents them from consulting their sexual problems.

## Discussion

The results of this study provide new insights into why older adults from a Central European country (Poland) might not seek help regarding their sexual problems. In line with other results published so far, this study’s findings confirm that only a small percentage of older individuals discuss sex-related concerns with their doctors (Moreira et al. 2008; Hinchliff and Gott 2011; Fileborn et al. 2017; Schaller et al. 2020); however, issues that constitute the main barriers identified in this study are somewhat different to previous research. The belief that sexual problems in older age are not real problems is present in the participants’ narratives and is consistent with older adults conforming to the “asexual old age” stereotype documented in the literature (Hinchliff and Gott 2011; Bauer et al. 2016). From this perspective, all examples of sexual decline are considered natural and irreversible, and, therefore, do not qualify for treatment (Moreira et al. 2008). However, this study’s results point to an even more detrimental way of perceiving sex-related issues: not recognising them as problems at all. This resonates with Hinchliff and Gott’s (2011) observation about the potential discrepancies between older adults, doctors, and researchers in defining sexual problems. One of the reasons for this might be current older adults’ insufficient knowledge regarding their sexual functioning, originating from poor sexual education and past societal taboos on sexuality during the Communist regime after the World War II (Hinchliff and Gott 2008; Mikołajczak and Pietrzak 2015). It is possible that in the cultures perceived as conservative—where sexual activity was centred around the male erection and sexual readiness—only related issues were considered real medical problems while others were disregarded (Hinchliff and Gott 2008; Sandberg 2013). It should be noted that some older adults, especially women, raised in such a context may lack basic sexual knowledge (e.g. that pain during intercourse or low sexual desire might be treatable), and for this reason may not seek treatment or support.

It has been repeatedly reported in the literature that embarrassment and a sense of inappropriateness in the face of a conversation with a doctor lead older adults to withdraw from contact (Gott and Hinchliff 2003; Corona et al. 2013; Fileborn et al. 2017). However, it seems that the main barrier for women and men in this study was not shame or embarrassment, but the anticipation of a doctor disregarding *any* “irrelevant” issue raised during the consultation. This is consistent with the reported opinions of healthcare professionals, who often perceive the sex-related issues of their older patients as irrelevant or less important than other medical problems (Gott et al. 2004; Malta et al. 2018; Gewirtz-Meydan et al. 2019). Yet, in the current study, this dismissive attitude was also experienced by older patients when introducing *non*-*sexual* concerns, which reinforced their reluctance to discuss *any* new problem during a consultation. These negative experiences with doctors, as recalled in the participants’ narratives, paint a picture of older adults being politely advised to “appreciate what they still have instead of asking for more”. Such an ageist approach—whether intended or accidental—seems to affect older patients’ health-related expectations (or rather lack of them) (Chrisler et al. 2016). This supports the findings of Fileborn et al. (2017) concerning the impact of doctors’ affirmative responses on the openness of older Australian adults when discussing sex in a healthcare setting. This further indicates that the doctors’ attitudes towards their patients’ concerns (attentive and empathetic versus insensitive and authoritarian) play a vital role in older adults’ decisions to introduce a sexual problem or not during a consultation (Hinchliff and Gott 2011; Gewirtz-Meydan et al. 2019; Schaller et al. 2020).

The majority of this study’s respondents believe that only a sexologist can address sexual problems. This is in line with an observation by Sarkadi and Resenqvist (2001) about older Swedish women who see sexual health issues as a specialist’s (gynaecologist), and not generalist’s (GP) domain. This finding is in opposition to some previous research in which older people saw GPs as the most appropriate professional to discuss sexual problems with (Gott and Hinchliff 2003b; Hinchliff et al. 2000). This apparent contradiction could be explained by the idea that some older adults perceive sexuality as a strictly private sphere of human life—the purview of their GP; while others see sexual problems as beyond the scope of general medicine (Mikołajczak and Pietrzak 2015; Sinković and Towler 2019). Individuals raised in societies where sex is a taboo may assume that discussing and treating sexual problems requires advanced expertise, training, and exceptional openness, which in their understanding can only be provided by specialists. This conviction is likely to be reinforced by GPs’ reluctance to initiate discussions on the topic. This should be considered a significant barrier to seeking treatment, since, in consequence, older adults do not see the readily available GP as a potential source of help (Hinchliff and Gott 2011; Bauer et al. 2016). Regardless of the specifics of healthcare systems, specialist doctors (sexologists in particular) are harder to reach than GPs, less available in health centres, and often operate in private settings. Therefore, considerable effort is required to make an appointment with a specialist (searching, referrals, cost, etc.) (Stirbu et al. 2011; Zhang et al. 2019). Being aware of these difficulties may decrease the willingness of older adults to seek treatment (Moreira et al. 2008), and reduce the already small number of those who, having overcome prejudices, fears, and embarrassment, are ready to talk to a doctor about their sexual problems.

Although studies conducted so far on the subject do not report notable gender differences, some variations in the accounts of older women and men have been observed (e.g. women reporting more ageist responses from healthcare professionals, men more easily receiving pharmacological support) (Fileborn et al. 2017; Hinchliff et al. 2020; Schaller et al. 2020). In the current study, no such differences were observed; the statements on the topic were convergent across genders, even though the sample was diverse in many aspects and the narratives notably differed in other areas (Gore-Gorszewska 2020). It might be the case that—when addressing sex-related problems—the stereotype of ‘asexual old age’ is so predominant in the highly conservative culture of Poland that it suppresses any possible gender differences observed in other contexts (Gore-Gorszewska 2020)”.

The findings presented here suggest that a lack of fundamental knowledge regarding the nature of sexual problems and potential treatment options prevents older adults from conservative sociocultural settings (Poland) from seeking professional help. Moreover, the doctors’ dismissive attitude, whether experienced or anticipated by older patients, seems to have a devastating effect on their willingness to introduce sex-related concerns during the consultation.

The trend of ageing of the European populations, coupled with sexual well-being recognised as an important aspect of quality of life regardless of age, makes sexual health in later life an objective for public health interventions. Since this topic remains largely disregarded in Poland (Tobiasz-Adamczyk et al. 2009), the experiences and reflections of the older Poles presented in this paper are intended to raise awareness of the existence of this issue. These study results may support policy makers in considering, planning, and implementing educational programmes for middle-aged and older adults on how (and where) to care for their sexual health and well-being, as well as in designing guidelines for healthcare providers on addressing sexual problems of their older patients.

### Limitations

Although the main strength of this study is that it provides potentially informative insights via the voices of older adults, several limitations should be considered. The chosen recruitment procedure (participant self-selection) may have resulted in a selection bias towards individuals who are more comfortable discussing sexual issues: the voices of less forthcoming older adults might be under-represented. The sample was exclusively heterosexual (despite this not being a recruiting requirement), and although it reflects the heteronormative social context of the Polish older generation, it should be noted that the experiences of non-heterosexual individuals might differ. Nevertheless, a purposive sample of older adults with a varied socio-demographic characteristic was recruited, suggesting the views presented here are not limited to a specific demographic. Since the majority of participants have never discussed sex-related issues with a doctor, future research may benefit from exploring the experiences of older adults who have successfully raised the topic during medical consultations.

### Conclusions

Searching for assistance with sexual problems remains uncommon among older adults. This may reduce the likelihood of maintaining—or achieving—a satisfying and healthy sexual life if an individual chooses to remain sexually active. On top of the help-seeking barriers already reported in the literature, several others were identified as predominant for this study’s participants, including: not recognising symptoms as related to sexual problems that deserve treatment, experiencing doctors’ dismissive attitude, and believing strongly that only sexologists can address sexual problems. The fundamental knowledge about the symptoms, sources of support, and potential treatment options should be available to all, as it may contribute to older adults’ decision whether—and in what form—to continue sexual activity in later life. Healthcare professionals, apart from taking the initiative to introduce sexual health topics during consultations, should also bear in mind how important to their older patients is their attitude—respectful and attentive towards the patients’ sexual and non-sexual concerns. Educational campaigns should be tailored for older, but also middle-aged adults from conservative countries who might have missed out on sexual education. Comparable problems and barriers should not be allowed to affect current middle-aged populations in the future.

## Electronic supplementary material

Below is the link to the electronic supplementary material.

Supplementary material 1 (PDF 171 kb)
