# Supplementary material for: “Why not ask the doctor?” Barriers in help-seeking for sexual problems among older adults in Poland
Source: Int J Public Health. 2020 Sep 4;65(8):1507–15. doi: 10.1007/s00038-020-01472-6 (PMC7588367; doi:10.1007/s00038-020-01472-6)
Supplement: Supplementary file 1 — Supplementary material 1 (PDF 171 kb) [file 38_2020_1472_MOESM1_ESM.pdf]

Table 1 Main coding categories and illustrative quotations

	Theme	Main Categories	Quotations
**1**	**Lack of knowledge: sexual problems**		
**1.1**	Not recognizing symptoms as sexual problems	Not knowing own body/ Not recognizing problems	*"How was I supposed to know what's normal and what's a problem? We weren't told back then. I've never discussed sex with anyone. It [sex] was something you do privately, with your husband, that's all." (F)* *"Sexual problems as in when having sex is problematic? So, when you're ill, or after a surgery or a stroke, of course you avoid sex then." (F)*
		There is only ED (erectile dysfunction)	*"[Sexual problems] in what sense? That I was not fully functional as a man? No. Never." (M)*
		Double standards	*"We had it coded deeply within us, that only men 'are allowed' to have problems like that-erection and all that. A woman must always be ready [for sex], no matter how she feels." (F)* *"Sexual problems? Yes, I had one–my wife didn't want to satisfy my needs. That was the main problem." (M)*
**1.2**	Symptoms as natural part of ageing	Normal in later life/ Acceptance	*"That's how the nature planned it. That's just life." (F)*
		Irreversible	*"Nothing can be done. When you're younger, everything works fine and you have sex; when you get old, you just don't. Finito. Just like you cannot go full speed on a highway driving an old car." (M)*
**2**	**Fear of doctor's disapproval**		
**2.1** **2.2**	General dismissive attitude towards older patients/ Doctors dismiss sexual concerns	'Don't ask, don't tell'/ Silent agreement	*"Is this a joke? Has a doctor ever asked me about my sexual health? Seriously? Anyway, I would never ask him. After all, he's my doctor." (M)*
		Strengthening the passive acceptance	*"Once–with my vaginal dryness–I tried to consult my gynaecologist. He looked at me and said, and I quote, 'well, it is normal at your age, Mrs X, don't expect miracles'. And he did nothing. Can you imagine how I felt?" (F)*
		Disregarding	*"When you raise a concern, which is not important according to them [doctors], they ignore it or 'politely' belittle it. Try to bring up a sexual issue and you will see–they would probably laugh out loud or, if more respectful, would probably smile and think 'Oh, bless her!' while changing the subject." (F)*
		Lack of empathy and understanding	*"There is no respect from healthcare professionals for older people. They don't have time, treat us mechanically [...] and certainly do not discuss sex-related problems with patients over 50, and definitely over 60. Because in their opinion sex at this age is merely a caprice." (M)*
		Imposed priorities/ Be alive vs quality of life	*"When a doctor sees a patient–and a 68-year old like me–the doctor wonders if he should still be alive or not, let alone have sex." (M)* *"They constantly tell you 'you're lucky to be alive and going, what else do you want?'. So eventually you stop asking for more, you*

			don't fight for the quality of life. Why should you? You feel like a burden to the healthcare system." (F)
		Paternalistic model	*"The doctor is the one who leads the consultation. They ask the questions and you answer, not the other way around. No room to argue." (M)*<br>*"'I know better what's good for you because I'm a doctor' – that's how it looks like." (F)*
**3**	**Lack of knowledge: medical services**		
**3.1**	Not knowing where to find adequate support	Never searching/ Never considering	*"Where to go with a sexual health concern? Well, if I can be honest, I have never thought about it. Because in general you don't talk about it. So now when you ask, my head is empty, no ideas." (M)*
		Confusion/ Frustration	*"I've searched everywhere and couldn't find the right person. I have doctors in five different health centres in the city, and there was not a single specialist [sexologist] in any of them! This is ridiculous and frustrating. What else was I supposed to do?" (M)*<br>*"I would say probably a psychologist, psychotherapist or physiotherapist, whatever they are called." (F)*
		Feeling lost/left alone	*"Maybe somewhere on the internet? Because in the normal, real places I haven't seen anything, any signs, adverts. I don't know, I don't use the internet." (F)*<br>*"You have to help yourself – either deal with it on your own or get used to it, there is no alternative. This is Poland, you know the reality." (M)*
**3.2**	Only a sexologist can help	Intimate/difficult topic	*"It is such a delicate matter, an intimate area of life, too subtle to discuss it with a regular doctor. It requires a lot of tactfulness and sensitivity, and expertise of course. GPs are not trained to deal with it." (F)*
		Area of expertise	*"I would say only a specialist, a sexologist can address these issues. That's why doctors specialise. It's logical: heart problems–cardiologist, eyes–ophthalmologist, sexual problems–sexologist." (M)*
		Partial associations	*"I would probably try to reach that one sexologist I know about, from TV, Lew-Starowicz, he's old and famous. Lucky for me that I don't have to, he's probably very busy." (M)*
		Availability/ Access	*"There are a lot of doctors, but their specialties are related to illnesses, not sex. They might be somewhere, but I haven't noticed. Sometimes you see these notices: psychologist here, gynecologist there. It's more for women, and I haven't seen one for men." (M)*<br>*"They must have private practices as you never see their offices in health centres. Well, I haven't." (F)*

F – female participant, M – male participant

Poland, 2019
